# The Novel Mas agonist, CGEN-856S, Attenuates Isoproterenol-Induced Cardiac Remodeling and Myocardial Infarction Injury in Rats

**DOI:** 10.1371/journal.pone.0057757

**Published:** 2013-03-01

**Authors:** Sílvia Q. Savergnini, Danielle Ianzer, Mariana B. L. Carvalho, Anderson J. Ferreira, Gerluza A. B. Silva, Fúlvia D. Marques, Antônio Augusto B. Peluso, Merav Beiman, Gady Cojocaru, Yossi Cohen, Alvair P. Almeida, Galit Rotman, Robson A. S. Santos

**Affiliations:** 1 Department of Physiology and Biophysics, Federal University of Minas Gerais, Belo Horizonte, MG, Brazil; 2 Department of Morphology, Federal University of Minas Gerais, Belo Horizonte, MG, Brazil; 3 Compugen Ltd., Tel Aviv, Israel; Max-Delbrück Center for Molecular Medicine (MDC), Germany

## Abstract

CGEN-856S is a novel Mas agonist. Herein, we examined the effects of this peptide on isoproterenol (ISO)-induced cardiac remodeling and myocardial infarction (MI) injury. We also sought to determine whether CGEN-856S activates the underlying mechanisms related to Mas receptor activation. Heart hypertrophy and fibrosis were induced by ISO (2 mg·kg^−1^·day^−1^) in Wistar rats. After a 7-day treatment period with CGEN-856S (90 µg·kg^−1^·day^−1^) or vehicle, the cardiomyocyte diameter was evaluated in left ventricular sections stained with hematoxylin and eosin, and immunofluorescence labeling and quantitative confocal microscopy were used to quantify the deposition of type I and III collagen and fibronectin in the left ventricles. MI was induced by coronary artery ligation, and CGEN-856S (90 µg·kg^−1^·day^−1^) or saline was administered for 14 days. The Langendorff technique was used to evaluate cardiac function, and left ventricular sections were stained with Masson’s trichrome dye to quantify the infarct area. Using Chinese hamster ovary cells stably transfected with *Mas* cDNA, we evaluated whether CGEN-856S alters AKT and endothelial nitric oxide synthase (eNOS) phosphorylation. CGEN-856S reduced the degree of ISO-induced hypertrophy (13.91±0.17 µm vs. 12.41±0.16 µm in the ISO+CGEN-856S group). In addition, the Mas agonist attenuated the ISO-induced increase in collagen I, collagen III, and fibronectin deposition. CGEN-856S markedly attenuated the MI-induced decrease in systolic tension, as well as in +dT/dt and -dT/dt. Furthermore, CGEN-856S administration significantly decreased the infarct area (23.68±2.78% vs. 13.95±4.37% in the MI+CGEN-856S group). These effects likely involved the participation of AKT and NO, as CGEN-856S administration increased the levels of p-AKT and p-eNOS. Thus, our results indicate that CGEN-856S exerts cardioprotective effects on ISO-induced cardiac remodeling and MI-mediated heart failure in rats through a mechanism likely involving the eNOS/AKT pathway.

## Introduction

Cardiac remodeling is an adaptive response to the pathogenesis of several heart diseases that interferes with the function and structure of the myocardium [1]–[3]. This structural remodeling process predisposes patients to an increased risk of adverse cardiac events, including myocardial ischemia, myocardial infarction (MI), arrhythmias, and sudden cardiac death [3]. A growing body of evidence indicates that the renin-angiotensin system (RAS) plays an important role in the development and progression of cardiac remodeling. Angiotensin (Ang) II, the main end product of the RAS cascade, stimulates the biosynthesis of cardiac extracellular matrix (ECM) proteins, leading to interstitial and perivascular fibrosis [4], [5], and cardiomyocyte hypertrophy [6]. On the other hand, numerous studies have proposed that the cardioprotective axis of the RAS composed by Ang-converting enzyme (ACE) 2, Ang-(1–7), and the Mas receptor counterregulates these Ang II actions in the heart [7]–[11]. Indeed, it has been reported that Ang-(1–7) reduces the growth of cardiac myocytes through activation of the G protein-coupled receptor (GPCR) Mas [12] and inhibits cardiac fibroblast-mediated collagen deposition [13]. Additionally, the hearts of Mas-deficient mice exhibit marked changes in ECM protein expression leading to a profibrotic profile accompanied by cardiac dysfunction [14], [15]. Grobe et al. [8], [16] demonstrated that Ang-(1–7) prevented cardiac fibrosis elicited by either deoxycorticosterone acetate (DOCA)-salt treatment or Ang II infusion, independent of blood pressure changes. Moreover, AVE 0991, a nonpeptide Ang-(1–7) analog, prevents the development of isoproterenol (ISO)-induced hypertrophy and collagen deposition [17]. Recently, He et al. [18] reported that AVE 0991 prevents Ang II-induced myocardial hypertrophy in a dose-dependent manner. Taken together, these findings indicate that the ACE2/Ang-(1–7)/Mas axis is involved in the prevention and attenuation of cardiac remodeling.

The evidence supporting the cardioprotective effects of the ACE2/Ang-(1–7)/Mas axis prompted us to search for novel Mas ligands. We used a computational biology discovery platform that utilizes machine learning algorithms designed to predict novel GPCR ligands cleaved from secreted proteins by convertase proteolysis, which are extracted from the Swiss-Prot protein database [19], [20]. The predicted ligands were synthesized and screened for activation of 152 GPCRs using calcium flux and cyclic adenosine monophosphate (cAMP) assays [20]. Two novel peptide ligands, P61S and P33V, displayed high specificity for Mas, eliciting calcium influx in Mas-overexpressing Chinese hamster ovary (CHO) cells [20]. Furthermore, P61S, designated CGEN-856S, does not activate either Ang II type 1 (AT_1_) or type 2 (AT_2_) receptors [20], [21].

We recently reported that CGEN-856S elicits nitric oxide (NO)-dependent vasodilation mediated by Mas in rat and mice aorta rings [21]. Additionally, picomolar concentrations of this peptide (40 pmol/L) induced an antiarrhythmogenic effect, as demonstrated by reduced incidence and duration of reperfusion arrhythmias in isolated rat hearts [21]. In addition, acute CGEN-856S administration produced a dose-dependent decrease in the blood pressure of spontaneously hypertensive rats (SHR) [21]. Importantly, the actions of CGEN-856S are inhibited by the Mas antagonist A-779 or by genetic deletion of this receptor [21]. In general, the data obtained following the use of this peptide resemble to those reported previously for Ang-(1–7) and AVE 0991. Thus, in the present study, we hypothesized that CGEN-856S might mimic the antihypertrophic and antifibrotic effects induced by Ang-(1–7) in rat hearts. To test this hypothesis, we evaluated the cardiac structure of ISO-treated and of infarcted rats. In addition, the effects of CGEN-856S administration on AKT and endothelial NO synthase (e-NOS) activation were investigated.

## Materials and Methods

### Ethics Statement and Animals

Male Wistar rats weighing 240−300 g were obtained from the animal facility of the Biological Sciences Institute of the Federal University of Minas Gerais. The experimental protocols were approved by the Ethics Committee in Animal Experimentation of the Federal University of Minas Gerais, Brazil (CETEA-UFMG), in accordance with the National Institutes of Health (NIH) Guidelines for the Care and Use of Laboratory Animals (protocol #149/10).

### Isoproterenol Induction of Hypertrophy

Osmotic mini-pumps (Alzet®, model 2001) containing CGEN-856S (90 µg·kg^−1^·day^−1^, 7 days) or saline were implanted subcutaneously (sc) under anesthesia (10% ketamine/2% xylazine, 0.1 mL/100 g, intraperitoneally [ip]). After recovery from anesthesia, the animals were divided into 4 groups: i) saline + vehicle (olive oil, 1 mL·g^−1^·day^−1^, sc, 7 days), ii) saline + ISO (2 mg·kg^−1^·day^−1^, sc, 7 days), iii) CGEN-856S + vehicle; and (iv) CGEN-856S + ISO. Moreover, as a positive control, an additional group of rats were treated with losartan (1 mg·kg^−1^·day^−1^, gavage, 7 days) + vehicle (olive oil, 1 mL·kg^−1^·day^−1^, sc, 7 days) or losartan (1 mg·kg^−1^·day^−1^, gavage, 7 days) + ISO (2 mg·kg^−1^·day^−1^, sc, 7 days). The final gavage and sc injection volumes were approximately 0.5 and 0.2 mL, respectively.

### Histological Analysis

At the end of the 7-day ISO treatment period, the rats were sacrificed by decapitation and the hearts were immediately removed. The left ventricles were fixed in 4% paraformaldehyde for 48 h at room temperature. The tissues were dehydrated by sequential washes with 70%, 80%, 90%, and 100% ethanol and embedded in Paraplast® X-tra Tissue Embedding Medium (McCormick Scientific). Transversal sections (6 µm) were cut starting from the base area of the left ventricle at 40-µm intervals and stained with hematoxylin and eosin for cell morphometry (n = 4 for each group). The cardiomyocyte diameter was evaluated in the tissue sections (3−4 for each animal) using an ocular micrometer calibrated with a stage micrometer adapted to a light microscope (BX 60, Olympus) at 100×magnification and analyzed using Image Pro Express software. Only cardiomyocytes cut longitudinally with the nuclei and cellular limits visible were used for analysis (an average of 15 cardiomyocytes for each slice). The diameter of each myocyte was measured across the region corresponding to the nucleus. Approximately 50 cardiomyocytes were analyzed for each animal.

### Immunofluorescence Analysis

Immunofluorescence labeling and quantitative confocal microscopy were used to investigate the distribution and quantity of type I and III collagen and fibronectin present in the left ventricles of the animals included in the ISO protocol (n = 4−5 rats/group). The hearts were enclosed in Tissue Tek OCT compound (Miles Scientific, Chicago, IL, USA), immediately frozen in liquid nitrogen, and stored at -80°C. Ventricular sections (7 µm) were obtained using a cryostat at -20°C, mounted on slides, fixed with ethanol for 10 min, and dried at room temperature. The slides were rehydrated with phosphate-buffered saline (PBS) for 10 min and incubated in blocking solution (1% bovine serum albumin [BSA] and 0.1% Tween 20 in PBS) at room temperature for 30 min. The sections were incubated overnight at 4°C with one of the following primary antibodies: rabbit anti-human type I collagen (1∶100, Rockland), rabbit anti-human type III collagen (1∶100, Rockland), or rabbit anti-human fibronectin (1∶200, Rockland). All antibodies were diluted with a 1∶10 dilution of blocking solution. After 4−5 PBS rinses, donkey anti-rabbit immunoglobulin G (IgG) conjugated with Alexa Fluor 488 (1∶200, Molecular Probes) and DRAQ5 (1∶1000, Biostatus) were added for 1 h in the dark at room temperature. Following PBS washes, the sections were mounted in 25% glycerol/75% PBS and viewed with a laser scanning confocal microscope (Zeiss 510 Meta). Optimal confocal settings (aperture, gain, and laser power) were determined at the beginning of each imaging session and then held constant during the analysis of all samples. For quantitative analysis of collagen I and III and fibronectin, we used ImageTool 2.0 image analysis software to measure the fluorescence intensity of the randomly selected images. The 12-bit images were captured and analyzed in the gray scale range of 0−255. The fluorescence intensity was calculated as an average of the area (i.e., the sum of the gray values of all pixels divided by the number of pixels in the area) and the values were recorded as arbitrary units.

### Myocardial Infarction Procedure

Under anesthesia with 10% ketamine/2% xylazine (0.1 mL/100 g, ip), the rats were placed in the supine position on a surgical table, tracheotomized, intubated, and ventilated with room air using a respirator for small rodents. Subdermal electrodes were placed for electrocardiography (ECG). The chest was opened by left thoracotomy at the fourth or fifth intercostal space. To expose the heart, a small retractor was used to maintain rib separation. After pericardial incision, the heart was quickly removed from the thoracic cavity and turned to the left to allow access to the proximal left anterior descending (LAD) coronary artery. A 4-0 silk suture was snared around the LAD and tightly ligated to occlude the vessel. To increase the survival rate of the animals, coronary ligation was performed on a more distal portion of the LAD. The heart was then replaced and the chest was closed with 4-0 silk sutures. Sham-operated rats were treated in the same manner, although the coronary artery was not ligated. After the surgical procedures, ECG tracings were obtained to confirm myocardial ischemia, i.e., ST-segment elevation and increased R-wave amplitude. Infarcted rats received CGEN-856S (90 µmg·kg^−1^·day^−1^) or vehicle (saline) administered through osmotic mini-pumps (Alzet®, model 2002) for 14 days. An additional group of infarcted rats received captopril (1 mg·kg^−1^·day^−1^, 14 days) through daily gavage. Fourteen days after infarction induction, the rats were sacrificed and cardiac function was evaluated.

### Isolated Heart Preparation

The animals (n = 7−8 rats/group) were decapitated 10−15 min after ip injection of 400 IU of heparin. The thorax was opened, then the heart was carefully dissected and perfused with Krebs-Ringer solution (KRS) containing (in mmol/L): 118.4 NaCl, 4.7 KCl, 1.2 KH_2_PO_4_, 1.2 MgSO_4_, 7 H_2_O, 2.5 CaCl_2_, 2 H_2_O, 11.7 glucose, and 26.5 NaHCO_3_. The perfusion fluid was maintained at 37±1°C with a pressure of 65−75 mmHg and constant oxygenation (5% CO_2_/95% O_2_). A force transducer was attached through a heart clip to the apex of the ventricles to record the contractile force (tension, g) on a computer by a data-acquisition system (Biopac System). A diastolic tension of 1.0±0.2 g was applied to the hearts. Electrical activity was recorded on ECG (Nihon Kohden) with the aid of 2 cotton wicks placed directly on the surface of the right atrium and left ventricle. Coronary flow was measured every 5 min by collecting and determining the volume of heart effluent during a 1-min interval. After 15−20 min of stabilization, the functional parameters (systolic tension, diastolic tension, ±dT/dt, heart rate, and coronary flow) were recorded for an additional 30-min period.

### Quantification of the Myocardial Infarct Area

At the end of the perfusion, the left ventricles (n = 6−8 for each group) were fixed in 4% Bouin’s fixative for 24 h at room temperature. The tissues were dehydrated by sequential washes with 70%, 80%, and 90% ethanol, 3 washes with 100% ethanol, and 3 washes with xylene, and then imbedded in paraffin. Transverse sections (6 µm) of the left ventricles were cut starting from the median area immediately below the left coronary artery ligation at 40-µm intervals and stained with Masson’s trichrome to quantify the infarct area. The infarct area was measured in 2 tissue sections (both at the median area, one proximal and the other distal to the coronary ligation of the left ventricle) of each animal. Images (40×magnification) were obtained using a JVC TK-1270/RGB microcamera. The built-in KS300 software built of a Kontron Elektronick/Carl Zeiss image analyzer was used for infarct area quantification using the image segmentation function. The data were expressed as mm^2^.

### Cell Culture and Western Blot Analysis

CHO cells (American Type Culture Collection) stably transfected with Mas and selected by neomycin (CHO-Mas) were serum-starved 3 h before all experiments. Cells were stimulated for 10 min in serum-free Dulbecco’s modified Eagle’s medium (DMEM) F-12 medium (Sigma-Aldrich, St. Louis, MO, USA) with Ang-(1–7) (10^−7^ mol/L and 10^−9^ mol/L) or CGEN-856S (10^−7^ mol/L and 10^−9^ mol/L) (Compugen Ltd., Israel). Optimal conditions, such as the duration of the incubation and peptide concentration, were chosen based on our initial concentration-response studies [22]. Untransfected CHO cells were used as controls and submitted to similar experimental conditions.

After the incubation period, the cells were washed with PBS to remove metabolic residues and most of the floating cells. The remaining cells were scraped into 180 µL of lysis buffer (50 mM Na_4_P_2_O_7_, 50 mM NaF, 5 mM Na_2_EDTA, 5 mM NaCl, 5 mM EGTA, 10 mM HEPES, 1% Triton X-100, and a specific EDTA-free inhibitor cocktail) for each cell culture flask (75 cm^2^). The lysate was transferred to a 1.5 mL tube, homogenized, centrifuged at 14000 rpm for 20 min at 4°C, and the supernatant was transferred for another tube. The protein concentration was assayed using the Bradford protein method. Sixty micrograms of protein were loaded on a 10% sodium dodecyl sulfate (SDS) polyacrylamide gel, electrophoresed, and transferred to a nitrocellulose membrane (Bio-Rad, Hercules, CA, USA). The membranes were blocked in 5% dry milk for 1 h and incubated overnight with one of the following primary antibodies at 4°C: total AKT (1∶1000, Cell Signaling Technology, Danvers, MA, USA), p-AKT Ser473 (1∶500, Cell Signaling Technology, Danvers, MA, USA), p-eNOS Ser1177 (1∶500, Cell Signaling Technology, Danvers, MA, USA), p-eNOS Thr495 (1∶500, Cell Signaling Technology, Danvers, MA, USA), glyceraldehyde 3-phosphate dehydrogenase (GAPDH) (1∶1000, Cell Signaling Technology, Danvers, MA, USA), and Mas (1∶500) [22]. The secondary antibody was added for 1 h at room temperature. Protein band detection was performed using the Odyssey scanning system (Li-Cor, USA) using Odyssey software. The results were quantified by densitometry (Odyssey software), normalized for the GAPDH or total AKT levels, and then the ratio of the experimental values to the control values was calculated.

### Statistical Analysis

Data were expressed as mean ± standard error of the mean (SEM). Histological data from each animal were obtained by averaging all values acquired in each tissue section. Statistical analysis was performed using one-way analysis of variance (ANOVA) followed by the Bonferroni post hoc test. Confocal microscopy data were expressed as the percentage of the mean gray value in relation to the maximum value acquired in the ISO-treated group of each imaging session and the statistical analysis was performed using an unpaired Student’s *t*-test followed by the Mann-Whitney *U* test. One-way ANOVA followed by the Newman-Keuls post hoc test was used to evaluate the cardiac function. *P*<0.05 was considered statistically significant.

## Results

**Figure 1:**
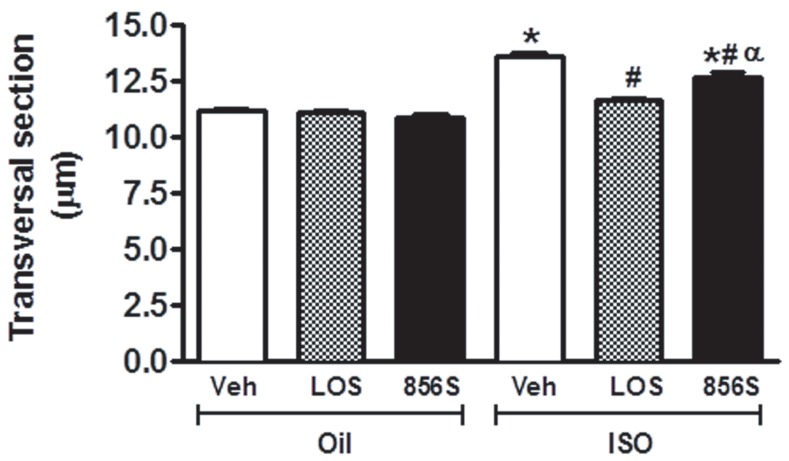
Effects of CGEN-856S and losartan administration on the cardiomyocyte diameters of isoproterenol-treated rats. Animals were treated with isoproterenol (ISO) for 7 days to induce heart hypertrophy or with olive oil as a control. The effects of CGEN-856S were compared to those of saline as a negative control (Veh) or losartan (LOS) as a positive control. Values are expressed as mean ± standard error of the mean (SEM), n = 4−5 animals. *P<0.05 vs. oil +Veh; ^#^ *P*<0.05 vs. ISO+Veh; ^α^ *P*<0.05 vs. ISO+LOS.

The cardiomyocyte cross-sectional area, a measure of cardiac hypertrophy, was significantly increased in ISO + vehicle-treated animals compared to oil + vehicle-treated rats (11.17±0.09 µm vs. 13.91±0.17 µm in ISO + vehicle-treated rats, Figure 1). CGEN-856S treatment reduced the degree of cardiac hypertrophy, as evidenced by a significant decrease in the cardiomyocyte diameter (13.91±0.17 µm vs. 12.41±0.16 µm in ISO + CGEN-856S-treated rats, Figure 1). Losartan administration also attenuated the ISO-induced increase in the cardiomyocyte diameter (13.91±0.17 µm vs. 11.63±0.08 µm in ISO + losartan-treated rats, Figure 1). CGEN-856S or losartan + oil treatment did not significantly affect the cardiomyocyte diameter.

**Figure 2:**
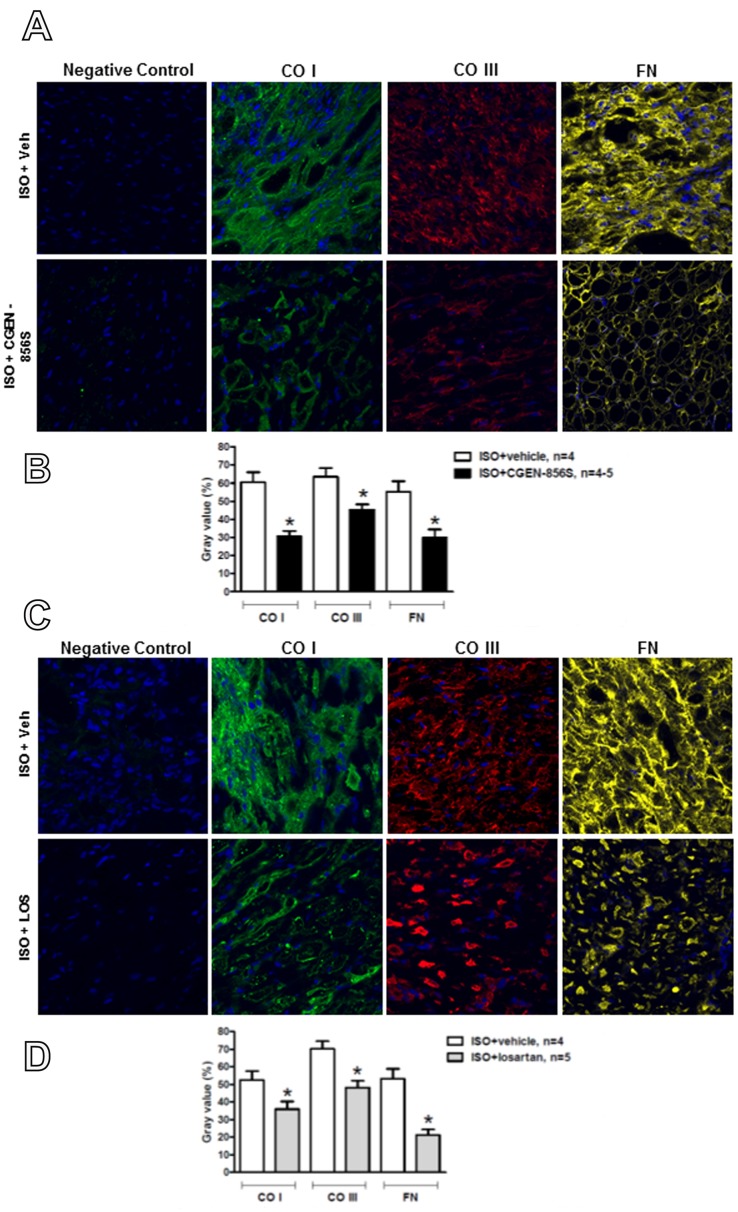
Effects of CGEN-856S and losartan administration on the deposition of type I collagen (CO I), type III collagen (CO III), and fibronectin (FN) in the left ventricles of isoproterenol (ISO)-treated rats. (A) Representative confocal photomicrographs and (B) quantification of CO I, CO III, and FN in the left ventricles of animals treated with CGEN-856S. (C) Representative confocal photomicrographs and (D) quantification of CO I, CO III, and FN in the left ventricles of animals treated with losartan. Values are expressed as arbitrary units (mean gray value ± SEM, n = 4−5 animals). *P<0.05 vs. ISO+vehicle.

To investigate the effects of CGEN-856S on cardiac fibrosis, we evaluated the deposition of cardiac type I and III collagen and fibronectin using immunofluorescence labeling and quantitative confocal microscopy. ISO administration resulted in a significant increase in the deposition of type I and III collagen and fibronectin (Figure 2). CGEN-856S treatment significantly reduced the deposition of type I collagen (60.57±5.52% vs. 30.63±3.00% in ISO+CGEN-856S-treated rats, Figures 2A and 2B), type III collagen (63.54±4.84% vs. 45.35±2.96% in ISO+CGEN-856S-treated rats, Figures 2A and 2B), and fibronectin (55.28±5.84% vs. 29.93±4.48% in ISO+CGEN-856S-treated rats, Figures 2A and 2B). Similar effects on cardiac fibrosis were obtained when ISO-treated rats were administered losartan (Figures 2C and 2D).

**Figure 3:**
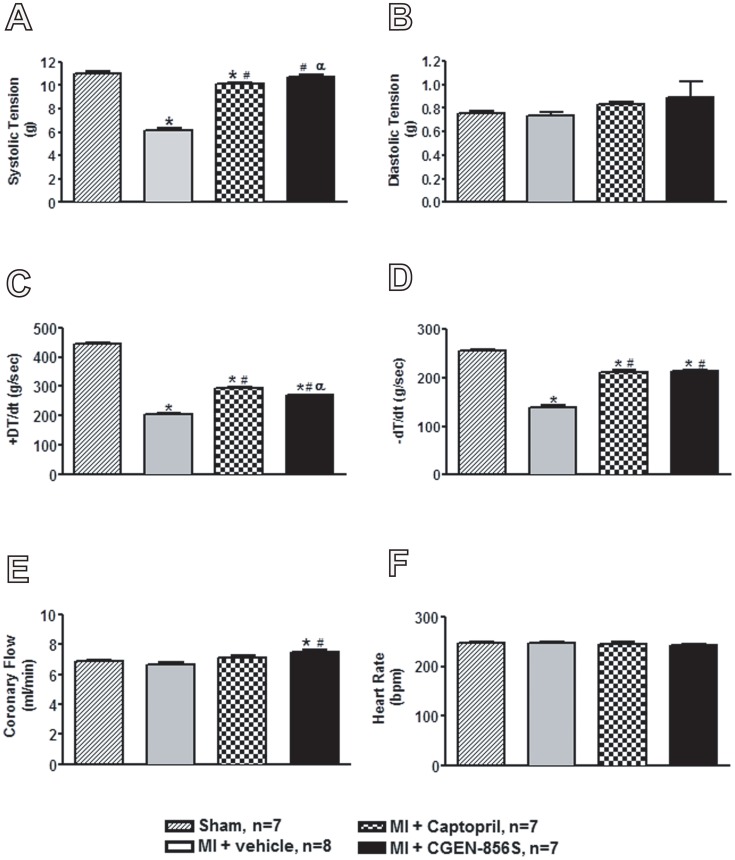
Effects of CGEN-856S and captopril administration on (A) systolic tension, (B) diastolic tension, (C) +dT/dt, (D) -dT/dt, (E) coronary flow, and (F) heart rate of rat hearts with myocardial infarction (MI). Values are expressed as mean ± SEM, n = 7−8 animals. *P<0.05 vs. sham; ^#^ *P*<0.05 vs. MI+vehicle; ^α^ *P*<0.05 vs. MI+captopril.

**Figure 4:**
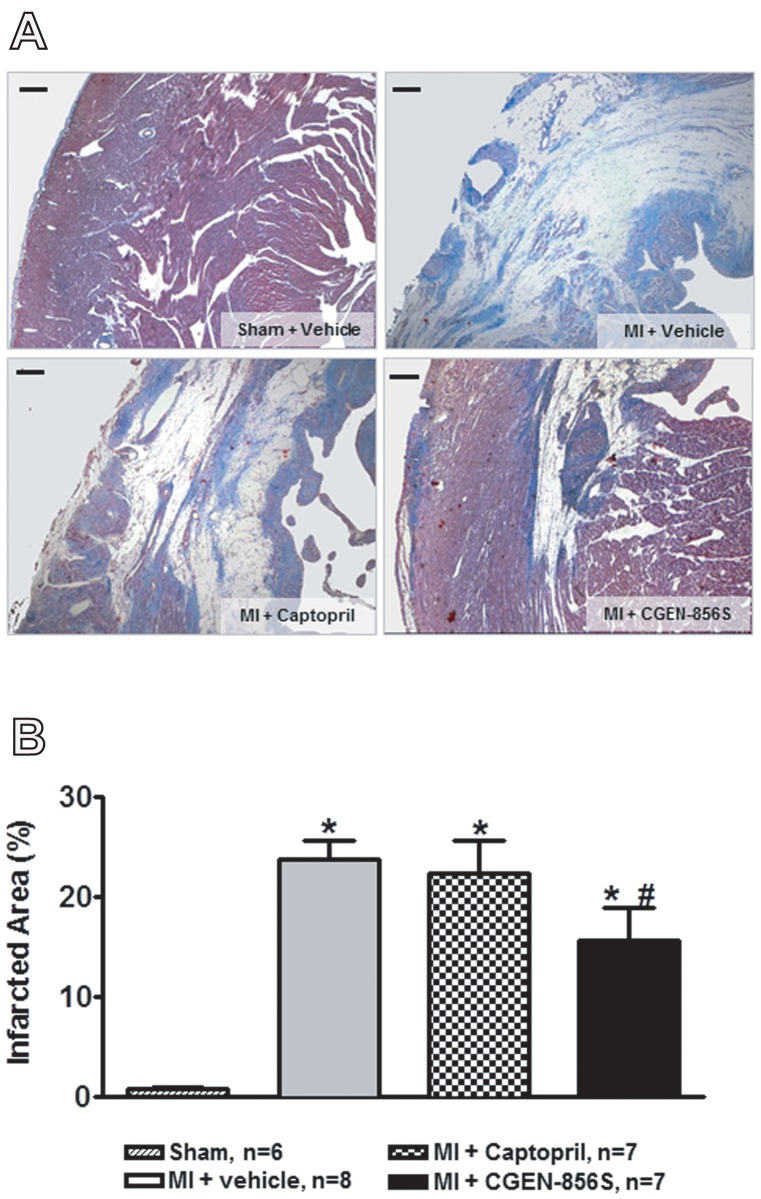
Effects of CGEN-856S and captopril administration on left ventricular infarct area. (A) Representative photomicrographs and (B) quantification of the infarct area of animals treated with CGEN-856S or captopril. Values are expressed as mean ± SEM, n = 7−8 animals. MI: myocardial infarction. *P<0.05 vs. sham; ^#^ *P*<0.05 vs. MI+vehicle.

The cardioprotective actions of CGEN-856S in the ISO model prompted us to study its effects on MI. As shown in Figure 3, MI resulted in reduced systolic tension and decreased velocities of contraction and relaxation (+dT/dt and -dT/dt, respectively) when compared to the sham-operated group. CGEN-856S treatment normalized the systolic tension and attenuated the decrease in the ±dT/dt induced by MI (Figures 3A, 3C, and 3D). In addition, CGEN-856S administration produced a slight but significant increase in the coronary flow when compared with infarcted rats (Figure 3E). No significant changes were observed in the diastolic function or heart rate among the groups (Figures 3B and 3F). The actions of CGEN-856S on the cardiac function of infarcted rats were similar to those effects observed in infarcted rats treated with captopril, an ACE inhibitor used as a positive control (Figure 3). Importantly, CGEN-856S treatment reduced the infarct area when compared to the vehicle-treated group (23.68±1.94% vs. 15.68±3.15% in MI+CGEN-856S-treated rats). In contrast, captopril did not induce any significant effect on the size of the infarct area (Figure 4).

**Figure 5:**
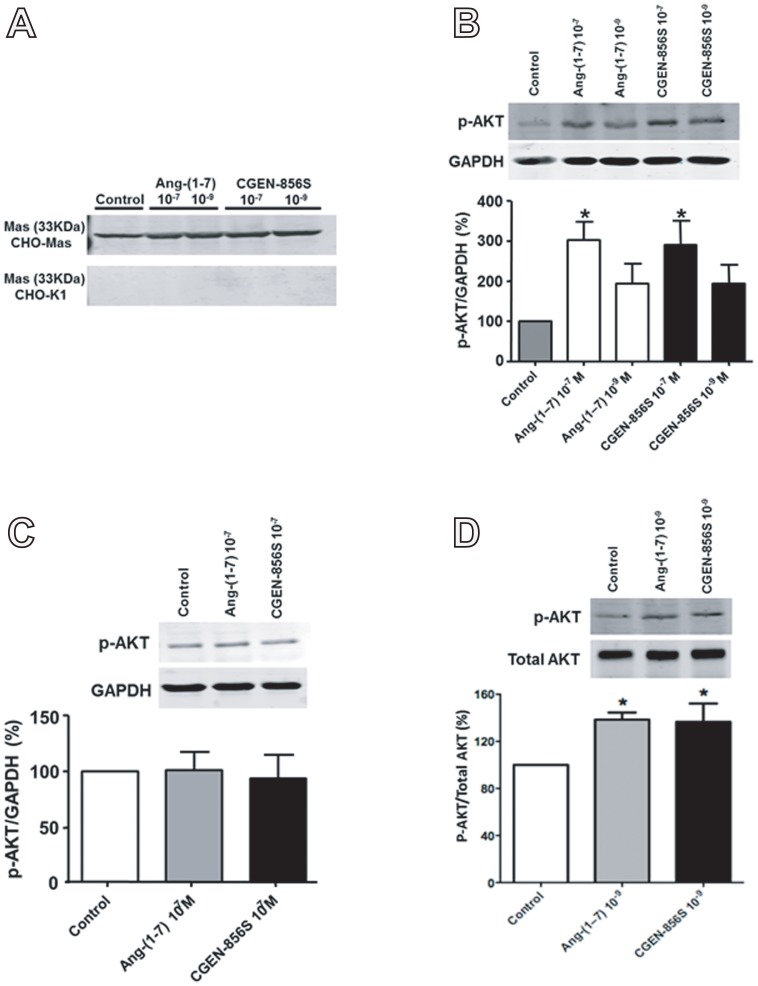
Effects of CGEN-856S and Ang-(1–7) administration on AKT phosphorylation and on the quantity of p-AKT. (A) Representative immunoblots demonstrating the presence of Mas in Mas-transfected CHO cells (CHO-Mas) and the absence of Mas in untransfected cells (CHO-K1). (B) Effects of CGEN-856S and Ang-(1–7) administration (10^−9^ and 10^−7^ mol/L) for 10 min on p-AKT levels in CHO-Mas cells. (C) The absence of effects of CGEN-856S and Ang-(1–7) administration (10^−7^ mol/L) for 10 min on p-AKT levels in CHO-K1 cells. (D) Effects of CGEN-856S and Ang-(1–7) administration (10^−9^ mol/L) for 5 min on AKT phosphorylation in CHO-Mas cells. Ang-(1–7) (10^−9^ and 10^−7^ mol/L) was used as a positive control and glyceraldehyde 3-phosphate dehydrogenase (GAPDH) and total AKT were used as loading controls. *P<0.05 vs. control. Results are expressed as the mean ± SEM of 4−6 experiments.

**Figure 6:**
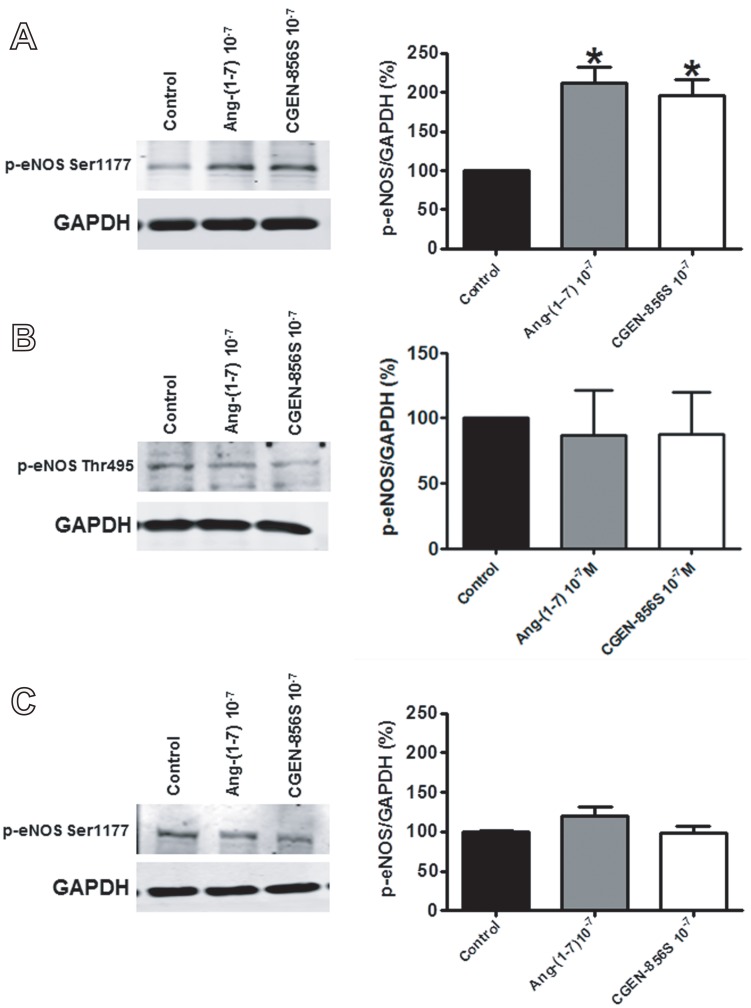
Effects of CGEN-856S and Ang-(1–7) administration on p-eNOS levels. CGEN-856S and Ang-(1–7) administration (10^−7^ mol/L) increased the quantity of (A) p-eNOS Ser1177, but not (B) p-eNOS Thr495 in CHO-Mas cells. (C) The absence of the effects of CGEN-856S and Ang-(1–7) administration (10^−7^ mol/L) on p-eNOS Ser1177 levels in CHO-K1 cells. Cells were exposed to the agonist for 10 min. Ang-(1–7) was used as a positive control and GAPDH was used as a loading control. *P<0.05 vs. control. Results are expressed as the mean ± SEM of 4−6 experiments.

In order to ascertain whether CGEN-856S stimulates similar intracellular pathways as Ang-(1–7), we assessed the effects of CGEN-856S administration on AKT phosphorylation and the level of p-AKT and p-eNOS in CHO cells transfected with Mas. As observed in Figure 5A, transfected CHO cells expressed Mas while untransfected CHO cells (CHO-K1) did not. After 10 min of stimulation, Ang-(1–7) and CGEN-856S (10^−7^ mol/L) significantly increased the level of p-AKT in CHO-Mas cells (Figure 5B). On the other hand, administration of 10^−7^ mol/L of CGEN-856S or Ang-(1–7) did not affect the level of p-AKT in untransfected CHO cells (Figure 5C). Of note, 10^−9^ mol/L of Ang-(1–7) and CGEN-856S also augmented AKT phosphorylation in CHO-Mas cells (Figure 5D). Furthermore, we observed that both Ang-(1–7) and CGEN-856S (10^−7^ mol/L) significantly increased the level of p-eNOS Ser1177 (Figure 6A), but not p-eNOS Thr495 (Figure 6B) in CHO-Mas cells. No significant changes in the level of p-eNOS Ser1177 were observed in untransfected CHO cells (Figure 6C).

## Discussion

The most important findings of the present study were that CGEN-856S, a novel Mas agonist, attenuated the cardiac damage induced by ISO treatment and MI. Specifically, CGEN-856S treatment prevented ISO-induced myocardial hypertrophy and fibrosis, improved heart function, and reduced myocardial injury in infarcted rats. CGEN-856S has been described as a specific Mas agonist with a >1000-fold lower affinity for AT_2_ receptors than Ang II. In addition, no evidence of CGEN-856S binding to AT_1_ receptors was observed [21]. Thus, we presented strong evidence that Mas activation using a specific agonist induces cardioprotective effects, as demonstrated in 2 distinct models of cardiac failure. Importantly, we were able to exclude any contribution of AT_2_ receptors to these effects, as the affinity of CGEN-856S for AT_2_ receptors is quite low.

CGEN-856S was first described as a Mas agonist by Shemesh et al. in 2008 [20]. The authors showed that this peptide elicits calcium influx in Mas-transfected CHO cells [20]. Additionally, we observed that CGEN-856S induces vasorelaxation through an NO- and Mas-dependent mechanism in rat and mouse aorta rings [21]. Further evidence of CGEN-856S binding to Mas was obtained by the observation that the Ang-(1–7) analogue D-Ala^7^-Ang-(1–7) (A-779) abolishes the vasorelaxant effects of CGEN-856S and by the effective CGEN-856S-induced displacement of fluorescent Ang-(1–7) [FAM-Ang-(1–7)] binding in Mas-transfected CHO cells [21]. Additionally, this vasodilative effect of CGEN-356S was absent in the aortic rings of *Mas*-knockout mice [21].

The antihypertrophic effect of Ang-(1–7) has been extensively reported. Transgenic rats [TGR(A1–7)3292] that possess a 2.5-fold increase in plasma Ang-(1–7) levels showed attenuated ISO-induced heart hypertrophy [11]. Tallant et al. [12] observed that Ang-(1–7) directly inhibits the growth of cultured cardiomyocytes through Mas activation. The antihypertrophic effects of Ang-(1–7) on Ang II-treated cardiomyocytes were prevented by N(G)-nitro-l-arginine methyl ester and 1H-1,2,4oxadiazolo4,2-aquinoxalin-1-one, suggesting that these effects are mediated by the NO/cyclic guanosine monophosphate (cGMP) pathway [23]. Furthermore, the nonpeptide analog of Ang-(1–7), AVE 0991, prevented Ang II-induced myocardial hypertrophy by putatively inhibiting the transforming growth factor (TGF)-β1/Smad2 signaling pathway [18]. In line with these findings, the novel Mas agonist, CGEN-856S, also reduced cardiomyocyte hypertrophy in rats challenged with ISO, a β-adrenergic agonist capable of inducing cardiac remodeling.

Another well-characterized action elicited by Ang-(1–7)/Mas is its antifibrotic effect. Grobe et al. [16] demonstrated that Ang-(1–7) selectively prevents collagen deposition in DOCA-salt rats. In an *in vitro* study, Ang-(1–7) inhibited collagen formation as measured by [^3^H]proline incorporation in cardiac fibroblasts of adult rats and decreased the mRNA expression of various growth factors, including *TGF-1*, endothelin-1, and leukemia inhibitory factor [13]. Nadu et al. [9] reported that the ISO-induced increase of type I and III collagen and fibronectin deposition observed in normal rats was attenuated in transgenic rats expressing an Ang-(1–7)-producing fusion protein [TGR(A1–7)3292]. In addition, it was reported that these transgenic rats are protected against cardiac dysfunction and fibrosis and show an attenuated increase in blood pressure after DOCA-salt treatment [24]. DOCA-salt [TGR(A1–7)3292] rats showed an important local increase in left ventricular Ang-(1–7) levels, which might have contributed to the reduced cardiac dysfunction and fibrotic lesions observed in these animals [24]. The compound AVE 0991 also decreased ISO-induced ECM protein deposition [17]. Thus, our current data are in agreement with previous studies using 2 well-known Mas agonists, Ang-(1–7) and AVE 0991 [25], [26]. These data corroborate the concept that Mas stimulation regulates cardiac remodeling and indicate that the cardioprotective effects of CGEN-856S are mediated by Mas activation.

Also, according to previous studies using Ang-(1–7) and the Mas agonist AVE 0991 [25], [26], activation of this receptor by CGEN-856S elicited significant improvements in cardiac function of infarcted hearts. CGEN-856S treatment restored the systolic tension, attenuated the MI-induced decrease in ±dT/dt, and increased the coronary flow as compared to control infarcted rats. The beneficial effects of Ang-(1–7)/Mas on cardiac function are one of the most important actions of ACE2/Ang-(1–7)/Mas axis activation. This includes both vascular and muscular effects [7], [11], [14], [15], [17]. Thus, the availability of compounds such as CGEN-856S that specifically activate Mas represents an important step toward translating these findings into clinical practice.

It is important to note that we previously demonstrated that CGEN-856S treatment produced merely a small decrease in the blood pressure of normotensive Wistar rats. This effect was observed only after infusion of higher doses of CGEN-856S (30 and 300 ng·kg^−1^·min^−1^). No significant changes in the heart rate were noted [21]. Thus, we believe that the functional and structural cardiac effects induced by CGEN-856S observed in our current study were not due to decreases in blood pressure.

We found that CGEN-856S stimulates AKT phosphorylation and increases the level of p-AKT and p-eNOS. These data suggest that, similarly to Ang-(1–7), CGEN-856S induces its beneficial effects through Mas activation via an eNOS/AKT-dependent pathway. Evidence for the involvement of Mas in the actions of CGEN-856S has been reported previously [21]. This new peptide induced NO-dependent vasodilation mediated by Mas in rat and mouse aorta rings [21]. Therefore, we hypothesized that CGEN-856S could trigger underlying mechanisms related to Mas stimulation. Consistent with our hypothesis, we observed that AKT and e-NOS were activated by CGEN-856S. Indeed, it has been reported that Ang-(1–7) elicits AKT phosphorylation in endothelial cells and cardiomyocytes [22], [27], [28]. It is important to note that high levels of NO/NOS might induce pro-oxidant effects. When NO reacts with superoxide, it generates the oxidant anion peroxynitrite (ONOO^−^), which provokes lipid peroxidation, nitrosation of amino acid residues, and disruption of cell membranes, cell signaling, and cell survival. Peroxynitrite also exerts proinflammatory actions [29]. Thus, a complete future study evaluating the effects of CGEN-856S on the oxidative balance of cardiomyocytes is warranted.

One may argue that 10^−9^ mol/L of CGEN-856S significantly increased AKT phosphorylation (ratio between p-AKT and total AKT, Figure 5D) but not the level of p-AKT (ratio between p-AKT and GAPDH, Figure 5B) in Mas-transfected CHO cells. However, this assertion is not true, as a careful analysis of the p-AKT level data (Figure 5B) reveals that CGEN-856S treatment almost doubled the quantity of p-AKT in CHO cells. Thus, we believe that these data did not achieve statistical significance merely due to the manner in which the data were organized, i.e., both concentrations of CGEN-856S (10^−9^ mol/L and 10^−7^ mol/L) were placed in the same graph (Figure 5B).

Of note, in contrast to Ang-(1–7), CGEN-856S presented no evidence of ACE inhibitory activity and showed low affinity to AT_1_ and AT_2_ receptors [21]. This suggests that the cardioprotective effects induced by CGEN-856S in these cardiac remodeling models might be independent of any action on ACE activity or other angiotensin receptors. Also, it is interesting to note that the CGEN-856S compound is more stable than Ang-(1–7) [21]. However, all of these possibilities warrant confirmation in cardiac tissues.

In summary, our current findings demonstrated that CGEN-856S treatment attenuates ISO and MI-induced heart damage likely through a mechanism involving the Mas/eNOS/AKT pathway. These data further support the protective role of Ang-(1–7) in the cardiovascular system and provide evidence that stimulation of this GPCR might be a potential therapeutic approach for cardiovascular diseases.
